# Test-retest reliability of FreeSurfer measures of neurodegeneration

**DOI:** 10.1016/j.neuroimage.2026.121920

**Published:** 2026-04-09

**Authors:** Henry Rusinek, Louisa Bokacheva, Haiyun Chen, Arjun Masurkar, Ricardo Osorio, Rebecca Betensky, Alok Vedvyas, Joshua Chodosh, Yongzhao Shao, Timothy Shepherd, Karyn Marsh, Thomas Wisniewski

**Affiliations:** a Department of Radiology and Psychiatry, NYU Grossman School of Medicine, 660 First Ave, New York, NY 10016, USA; b Department of Neurology, NYU Grossman School of Medicine, 145 East 32nd Street, New York, NY 10016, USA; c Department of Psychiatry, NYU Grossman School of Medicine, 145 East 32nd Street, New York, NY 10016, USA; d Department of Medicine, NYU Grossman School of Medicine, 550 First Ave, New York, NY 10016, USA; e Department of Biostatistics, NYU School of Global Public Health, 708 Broadway, New York, NY 10003, USA; f Department of Population Health, NYU Grossman School of Medicine, 550 First Ave, New York, NY 10016, USA

## Abstract

Reliable structural brain measurements are essential for studying neurodegeneration and for designing adequately powered aging and Alzheimer’s disease (AD) research. We evaluated the test–retest reliability of FreeSurfer 7.1 morphometric measures in 100 older adults (mean age 73.5 years) ranging from cognitively unimpaired to dementia. Each participant underwent two T1-weighted 3T MRI scans on the same scanner within a short interval (mean 5.5 weeks), minimizing biological change. Segmentation was performed in both standard cross-sectional and longitudinal FreeSurfer modes, focusing on AD-relevant volumes of entorhinal cortex, hippocampus, lateral ventricles, choroid plexus, and the AD cortical thickness signature. Reliability was quantified using absolute and root-mean-square test–retest differences, standard deviation of differences, and intraclass correlation coefficients. Longitudinal processing improved precision by 15–50% across most measures compared with cross-sectional processing, with the largest gain observed for entorhinal thickness. Larger, anatomically well-defined regions (e.g., hippocampus, AD signature) demonstrated higher reliability than small structures or those with complex geometry (e.g., entorhinal cortex, choroid plexus). Image quality, indexed by the Euler characteristic, was the only factor significantly associated with measurement variability; reliability was unrelated to age, sex, cognitive status, inter-scan interval, or amyloid/tau PET burden. Power analyses indicated that detecting a 1% within-individual change requires sample sizes ranging from 36 (AD signature) to >300 (entorhinal cortex). We observed low reliability of choroid plexus volumetry by FreeSurfer 7. These results provide practical benchmarks for expected FreeSurfer measurement variability in older adults. They highlight the advantages of longitudinal processing and rigorous quality control for research on brain aging and AD.

## 1. Introduction

Automated brain segmentation from MR images is widely used in neurologic and psychiatric research for analyzing brain changes in healthy aging and pathological states (Bonarota et al., 2025; Morra, 2008). Other medical applications include quantification of brain function through multimodal imaging (e.g., combined PET and MR imaging), surgical planning, image-guided interventions, and even a routine radiological diagnosis (Catana, 2012). An increasingly important task is the detection and monitoring of neurodegeneration by quantifying brain atrophy in regions vulnerable to changes associated with Alzheimer’s disease (AD) and related dementias (ADRD) (Bonarota, 2025; Young, 2020). Segmentation and cortical parcellation are most often applied to T1-weighted (T1W) pulse sequences which combine good tissue contrast with sub-millimeter isotropic spatial resolution.

FreeSurfer, an open-source software from the Martinos Center for Biomedical Imaging, is one of the widely used brain segmentation software packages (Fischl, 2012). FreeSurfer uses a brain atlas as a template to automatically label multiple brain structures on T1W MRIs and to estimate their volume, thickness, surface area, and curvature.

Accuracy and reliability (precision) are of utmost importance in interpreting results of morphometric software such as FreeSurfer. Reliability is best estimated experimentally. Depending on the type of study (single center vs multi-site), one may be interested in either measurement repeatability or reproducibility. Repeatability refers to the consistency of results after repeating the measurement under identical procedural settings. Reproducibility reflects the resilience of results to variable equipment or acquisition parameters. Knowledge of measurement reliability is essential for statistical power analysis and for interpreting observed differences between groups or longitudinal changes within individuals.

FreeSurfer reliability of brain segmentation has been explored mainly in younger adults (Haddad, 2023; Hedges et al., 2022; Knussmann, 2022). The reliability of AD imaging biomarkers in the older population has been less studied (Jack, 2024). This distinction may be relevant, as there are reports that adults older than 65 are more likely to move more during MR image acquisition than those aged 20–40 (Madan, 2018). It has been reported that head motion is also larger in patients with cognitive impairment than in unimpaired adults (Haller, 2014), which could confound brain aging studies (Pardoe, 2016; Reuter, 2015).

To assess the repeatability of FreeSurfer segmentation in older adults, we used two T1W 3T MRIs, acquired on the same scanner within a relatively short time interval, in 100 subjects from a cohort encompassing both healthy aging and cognitive decline. Segmentation was performed using FreeSurfer 7.1 in both cross-sectional and longitudinal modes (see Methods for details). We also analyzed factors that could have an impact on segmentation reliability (Bedford, 2023; Hedges et al., 2022; Rosen, 2018).

## 2. Methods

### 2.1. Participants

MRI and other data were obtained from a longitudinal aging cohort that is part of the NIH/NIA-sponsored NYU Alzheimer’s Disease Research Center (ADRC) located in New York City. The full cohort consists of 484 well-characterized individuals aged 65–97 (mean age 73.2), 64% female, all without major organ disease or significant non-AD/ADRD neurological disorders. Participants undergo detailed psychometric testing, health surveys, and neurological examinations in accordance with the National Alzheimer’s Coordinating Center Uniform Data Set. They provide blood samples from which AD/ADRD plasma biomarkers are derived. Subjects also undergo brain imaging, including MRI, amyloid PET and tau PET. Cognitive status ranges from cognitively unimpaired to dementia, stratified via the Global Deterioration Scale (GDS) and the Clinical Dementia Rating (CDR) (Hughes, 1982; Reisberg, 1982). Diagnoses are made via consensus conference. The racial and ethnic composition (56.6% White, 32% Black, 11.4% Hispanic) represents the demographics of the New York City area.

We included data from 100 consecutive participants who satisfied the following criteria:

- two brain MRI examinations;
- the first MRI date after January 2022;
- <180 days between the two MRIs (i.e., short enough to minimize the likelihood of structural brain changes);
- availability of both amyloid and tau PET examinations.

All participants provided written informed consent prior to imaging as part of their cohort participation. The ADRC study was approved by the Institutional Review Board of NYU Langone Health.

### 2.2. MRI protocol

All images were acquired on the same Siemens Biograph 3T PET-MR system using a 20-channel head coil. The protocol included a sagittal T1-weighted MPRAGE sequence (TR = 2300 ms; TE= 2.98 ms; TI = 900 ms; flip angle = 9°; bandwidth = 240 Hz/pixel; acceleration factor = 2; matrix = 256 × 240 × 208; voxel size = 1 × 1 × 1 mm^3^; acquisition time = 5:12 min). This widely used sequence is included in several large multisite projects such as ADNI (https://adni.loni.usc.edu) and SCAN (https://scan.naccdata.org/). Head positioning followed routine clinical procedures, including laser-guided centering, alignment to minimize tilt, neutral neck positioning, and instructions to remain still.

### 2.3. Amyloid and tau PET

Amyloid PET was acquired using the 18F-florbetaben (FBB) tracer for 30 min at 90–120 min after an injection of 300 MBq (8.1 mCi) of FBB. The standardized uptake value ratio (SUVR) for FBB was derived using a composite neocortical region of interest encompassing the frontal, lateral parietal, and lateral temporal cortices, with the whole cerebellum designated as the reference region (Landau, 2021). Tau PET used 18F-MK6240 tracer (185 MBq) with uptake measured 90–120 min post-injection. SUVR was derived from a composite target region including the fusiform gyrus, parahippocampal gyrus, hippocampus and posterior cingulate. Cerebellar gray matter served as the reference region (Bullich, 2022; Pascoal, 2020).

### 2.4. Measures of neurodegeneration

We focused on volumes and cortical thickness of brain regions vulnerable to AD/ADRD-related change (Young, 2020). The total intracranial volume (ICV) was used for normalization. These regions included the entorhinal cortex, hippocampus, lateral ventricles and choroid plexus (Jack, 2024). We also included the AD signature (Dickerson, 2009), defined as the mean thickness across the following bilateral regions: middle and inferior temporal gyri, temporal pole, superior and inferior parietal lobules, superior and middle frontal gyri, supramarginal gyrus, entorhinal cortex, fusiform gyrus, and precuneus. The AD signature is a validated biomarker used to detect anatomic abnormalities consistent with preclinical AD (Busovaca, 2016; Clauss, 2025; Fletcher, 2021). Enlargement of choroid plexus has recently been linked to memory and cognitive decline and other AD-related symptoms (Jiang, 2024; Sun, 2025).

In the initial run, the first and the second examinations were processed separately using *recon-all* command. We then repeated processing in longitudinal mode using the *recon-all -long* option, introduced in recent versions of FreeSurfer. In this mode, all exams from the same individual are processed in tandem using the same initial approximation of brain surfaces (Reuter, 2012). Longitudinal mode aims to provide an unbiased analysis of brain changes over time, such as those associated with disease progression or intervention.

### 2.5. Reliability measures

Given the first and the second estimates $m_{\text{i,p}}$, ${m^{’}}_{\text{I,p}}$ of metric i for participant p, we assessed reliability using the mean absolute difference, $\text{avg}(|d_{\text{i,p}}|)$, $d_{\text{i,p}}=m_{\text{i,p}}-{m^{’}}_{\text{I,p}}$, standard deviation of the difference ${\mathrm{SD}}_{i}=\mathrm{sd}\;(d_{\text{i,p}})$, root mean square difference ${\mathrm{RMSD}}_{i}$, and intraclass correlation coefficient, ICC_i_. ${\mathrm{SD}}_{i}$ is used for power analysis based on paired t-test, i.e., for estimating the sample size required to detect a specified change (Chow, 2007). ${\mathrm{SD}}_{i}$ reflects only random variability and ignores systematic bias. ${\mathrm{RMSD}}_{i}$, on the other hand, captures total disagreement between measurements, including both random variability and systematic bias. These are related by: ${{RMSD}_{i}}^{2}={{SD}_{i}}^{2}+{{\bar{d}}_{i}}^{2}$, where the bias ${\bar{d}}_{i}=avg\left(d_{i,p}\right)$ is the mean difference of metric i between test and retest. When systematic bias is negligible ($\bar{d}\approx 0$), SD ≈ RMSD. The intraclass correlation coefficient (ICC, range from 0 to 1) provides an additional measure of reliability (Barchard, 2012). We report 95% confidence intervals for ICC. Reliability measures were computed separately for the standard cross-sectional and longitudinal FreeSurfer modes.

We tested whether a composite of inter-exam differences for five key neurodegeneration metrics was associated with demographic, cognitive or imaging characteristics. The five measures were entorhinal cortex thickness, AD signature, and the volumes of the whole hippocampus, lateral ventricle and choroid plexus. For each metric we computed the normalized absolute difference $e_{i,p},e_{i,p}=\frac{\left|m_{i,p}-{m^{\prime }}_{i,p}\right|}{{\bar{m}}_{i,p}}$, a *subject-level* reliability estimate, *where* ${\bar{m}}_{i,p}$ is the mean of the two observations. We then calculated the test–retest variance across participants for each metric and used the inverse variances as weights:

$$w_{i}=\frac{1}{Var\;\left(e_{i,p}\right)}.$$

The weighted $e_{i,p}$ were combined to produce a composite score $c_{p}$ that accounts for scale and variability differences across measures:

$$c_{p}=\frac{1}{\bar{w}}\sum_{i}e_{i,p}w_{i},\bar{w}=\sum_{i}w_{i}.$$

We tested associations between $c_{p}$ and age, sex, cognitive status (GDS), inter-exam interval, amyloid and tau PET levels, and image quality (Euler characteristic). Linear regression was used for continuous predictors, t-test for sex differences, and analysis of variance for GDS. All analyses were conducted using R version 4.2.2.

Image quality was quantified using the Euler characteristic of the initially fitted surfaces (Hatcher, 2002; Worsley, 1996), a value that reflects the number E of unanticipated holes and “handles” in the triangular mesh representing the pial and white-gray matter surfaces. In our sample, E had a mean of 43 ± 39 (range 7–243). Although FreeSurfer 7 pipeline attempts to remove these defects during the cleaning stage, we used the pre-cleaning E values, averaging across hemispheres and across two MRI acquisitions.

For paired t-test power analysis, the required sample size n can be approximated using Eq. 3.1.2 in (Chow, 2007):

$$\sqrt{n}=\frac{s}{\theta }(Z(1-\alpha /2)+Z(\beta )),$$

where s is the standard deviation of the paired differences in volume, expressed in raw volumetric units, θ is the hypothesized difference we seek to detect, also in raw volume units, Z(⋅) is the standard-normal quantile function, α is the significance level and β is the desired power.

## 3. Results

**Fig. 1.**
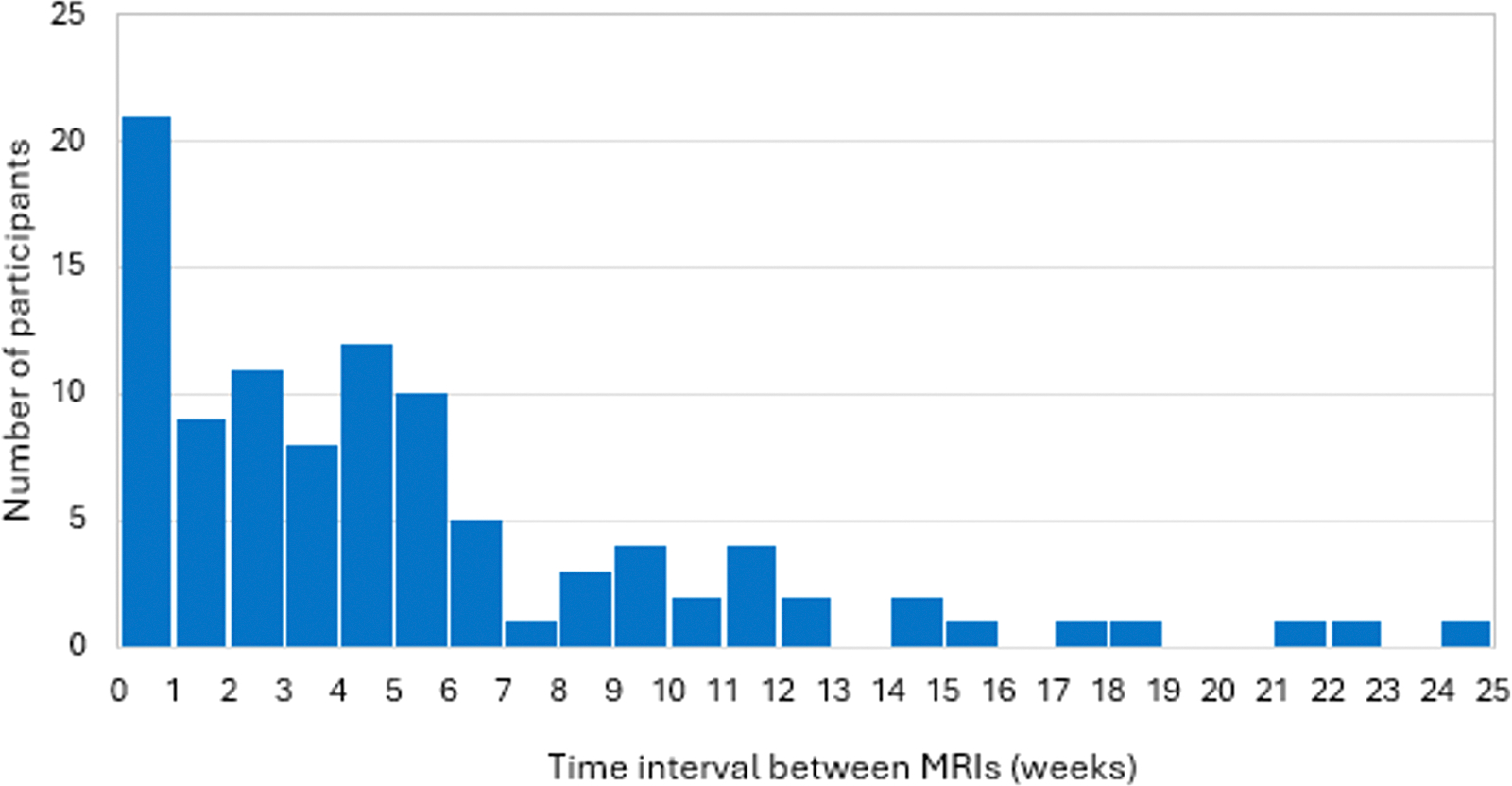
Distribution of time intervals between the initial and the second MRI exam.

The 100 participants (mean age 73.5 ± 6.1 years, 64% women, 33% Black, 16.7 ± 2.4 (range 9–20) years of education) underwent two MRIs separated by an interval of 5.5 ± 5.2 weeks (Fig. 1). The distribution of GDS was: 30 cognitively unimpaired, 46 with subjective cogntive impairement, 24 with mild cognitive impairment or AD.

**Fig. 2.**
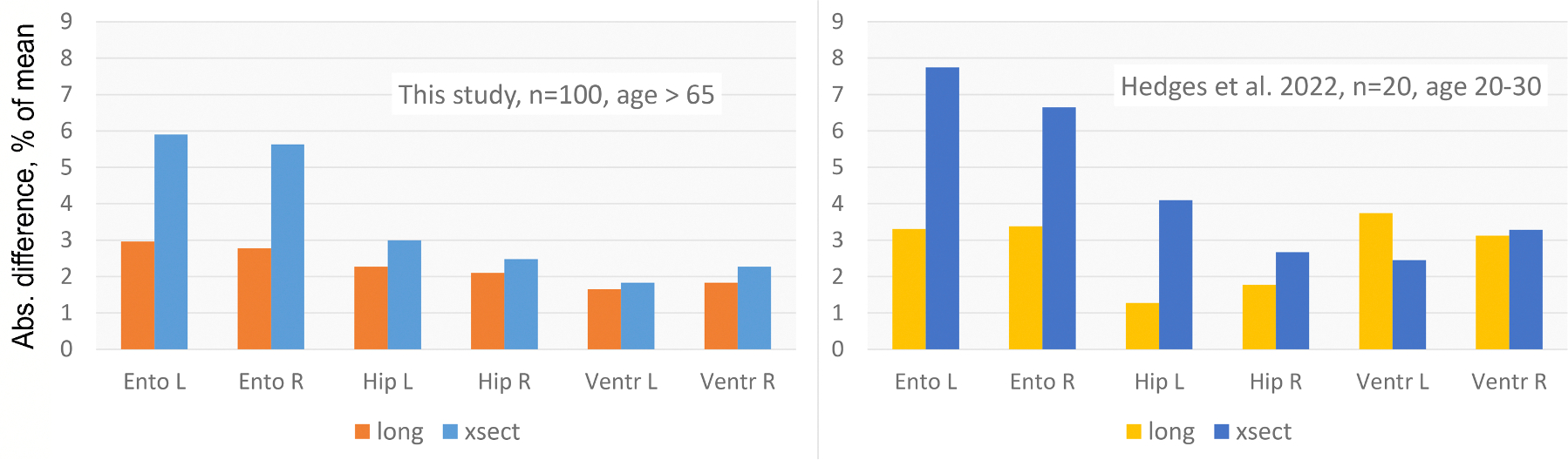
Test-retest reliability for cross-sectional and longitudinal analyses. Bars represent the average of absolute differences |MRI2−MRI1|/0.5*(MRI1+MRI2), in percents. (Left panel): in our sample the longitudinal analysis reduces the test-retest error by 50% for entorhinal cortex thickness and by ~10–20% for hippocamal and ventricule volume. (Right panel): corresponding estimates reported in supplemental data (eTables 1 and 2, columns “Three weeks, A1 vs B1”) by Hedges et al. (2022) for a different magnet and healthy young individuals.

Tables 1 and 2 report FreeSurfer reliability measures for the cross-sectional and longitudinal processing. The longitudinal pipeline generally demonstrated 15%–50% higher reliability (Fig. 2, left panel).

**Fig. 3.**
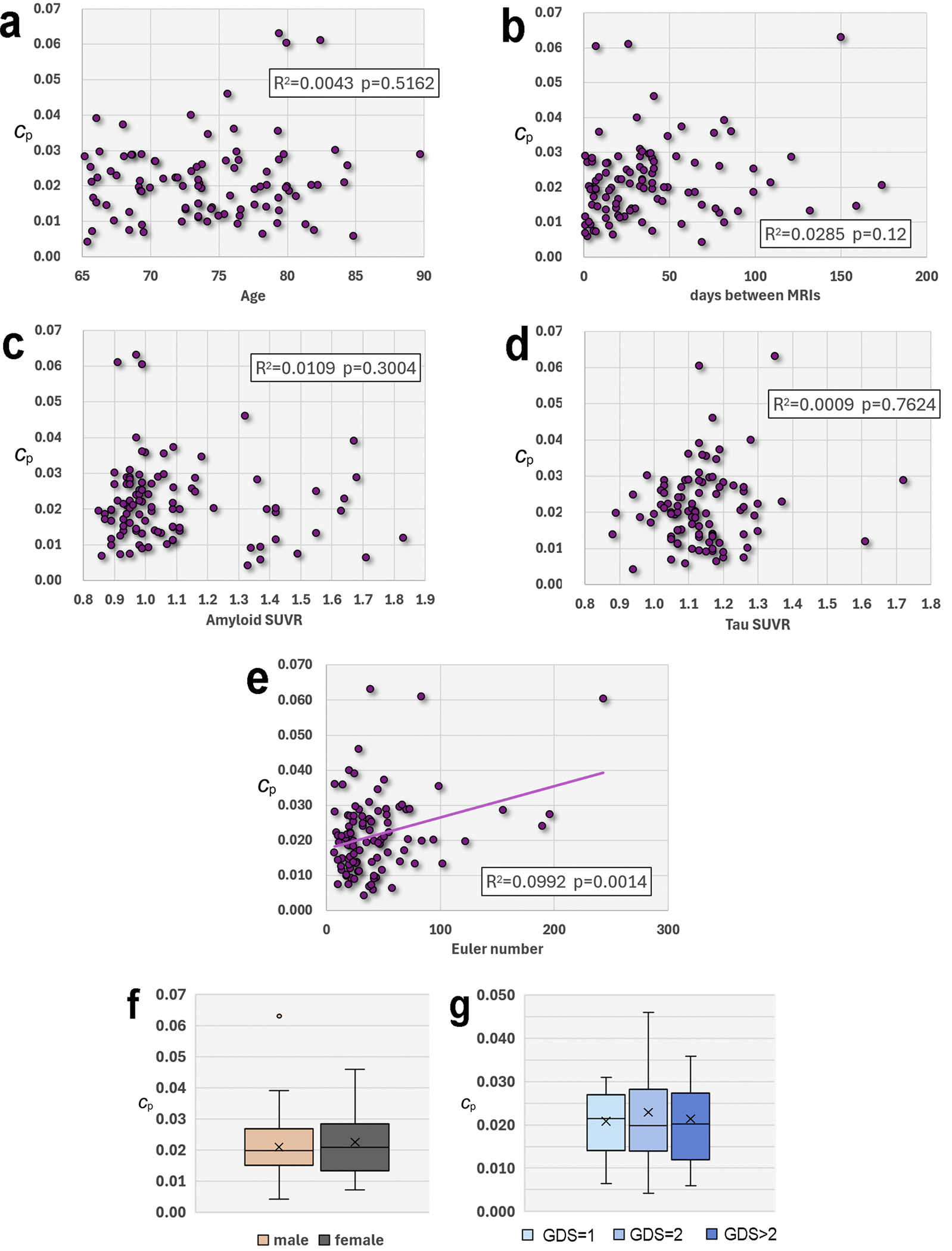
Associations between the composite FreeSurfer reliability score c_p_ and age, interval between MRI, amyloid and tau SUVR, and Euler number (a–e) and group comparisons by sex and GDS (f, g).

No associations were observed between the composite FreeSurfer reliability score $c_{p}$ and the inter-MRI interval or participants’ demographic, cognitive, or neurobiological characteristics (Fig. 3a–d, f–g). Specifically, testing the association between $c_{p}$ vs age we have R^2^ = 0.0043, p = 0.5162; $c_{p}$ vs interval between two MRIs R^2^ = 0.0285, p = 0.1200; vs amyloid SUVR R^2^ = 0.0109, p = 0.3004; vs tau SUVR R^2^ = 0.0009, p = 0.7624. The t-test (panel 3f) shows $c_{p}$ to be the same for male 0.0210 ± 0.0107 and female participants 0.0225 ± 0.0117, t=−0.670, df=98, p = 0.504. ANOVA results (panel 3 g) demonstrate no differentiation between $c_{p}$ across GDS=1, GDS=2 and GDS≥3 subgroups, df=2, F value = 0.269, p = 0.765. However, image quality—as expressed by the Euler number—showed a significant effect, R^2^ = 0.0992, p = 0.0014 (Fig. 3e).

Table 3 shows the sample sizes needed to achieve 80% power (α=0.05) to detect a 1% within-individual change in each brain measure using paired t-tests. For example, detecting a 1% change in the AD signature, whole hippocampus, or entorhinal cortex would require 36, 69, or 339 participants, respectively. In contrast, due to the low reliability of FreeSurfer segmentation of the choroid plexus, detecting small changes in choroid plexus volume is not feasible.

## 4. Discussion/conclusions

We evaluated the test–retest reliability of FreeSurfer 7.1 morphometric measures in an older cohort spanning a wide range of cognitive function. Our findings demonstrate that reliability varies across structures and depends on both region size and geometric complexity. Larger, well-defined regions such as the whole hippocampus and AD signature showed higher reliability than smaller structures like the entorhinal cortex and structures with intricate borders such as the choroid plexus. These observations are consistent with previous reports (Hedges et al., 2022; Jack, 2024; Reuter, 2012).

Comparisons between ICV–normalized and raw measures in cross-sectional mode show that normalization slightly reduces reliability, i.e., increases test-retest differences by approximately 10%. For instance, the RMSD% for hippocampal volume was 2.91%, compared to 3.21% for volume normalized to ICV. This effect is expected, as normalization propagates measurement error from both regional volumes and total ICV estimates. Nevertheless, normalization remains valuable for reducing confounding effects and improving comparability across individuals. Also note that there is no loss of reliability due to ICV normalization in longitudinal mode, as here the cranial cavity is assumed to be constant across time points.

The longitudinal FreeSurfer mode substantially improved precision compared to the standard mode across nearly all measures. This improvement was most pronounced for entorhinal cortex thickness, with error reduced by approximately 50%. These results align with earlier reports demonstrating that longitudinal processing reduces random variability by leveraging within-subject anatomical consistency over time (Hedges et al., 2022; Reuter, 2012). We therefore recommend the longitudinal pipeline for studies tracking subtle within-individual changes, such as disease progression or treatment effects.

Because the short test–retest interval minimizes true biological change, the within-individual variability in Tables 1–2 primarily reflects the measurement precision of the FreeSurfer 7 pipeline. The representative power calculations in Table 3 therefore illustrate the minimum sample sizes needed to detect a specific (arbitrary but realistic) hypothesized 1% volume change.

Hedges et al. (2022) tested reliability of FreeSurfer v7.1 and earlier versions using a 3T GE MR750 Discovery system with MPRAGE sequence in 20 healthy 20–30 years old adults. Their reported reliability patterns—including the superiority of the longitudinal mode and dependence on image quality—broadly agree with our findings. Although that study did not focus on aging or dementia, the supplementary data allowed a direct comparison of entorhinal cortex, hippocampus and ventricular measures. Fig. 2 compares normalized absolute differences for measures taken 3 weeks apart (A1 v s B1 scheme in Hedges et al. (2022)). The test-retest differences observed by Hedges et al. were approximately 25% larger than those in our study. This discrepancy should be interpreted cautiously, as the studies differed in scanner hardware, acquisition protocols, patient positioning and other experimental conditions that may influence segmentation variability. The results of both studies highlight similar reliability patterns across structures. Both Hedges et al. and the multisite study of Haddad et al. (Haddad, 2023) also demonstrate substantial reliability improvement in FreeSurfer version 7 relative to earlier versions.

This study is limited to data acquired on a single 3T Siemens system using a single T1-weighted MPRAGE sequence from the current ADNI protocol. While this restricts generalizability, the selected sequence is standard in aging and dementia research. We also did not compare FreeSurfer with alternative software packages, something that should be considered for future studies. Furthermore, we did not compare FreeSurfer 7 longitudinal processing stream with deformation-based approaches such as Jacobian Integration (Boyes, 2006; Tustison, 2019), Tensor-based Morphometry (Hua, 2013; Yushkevich, 2010) or Boundary Shift Integral (Leung, 2012; Rusinek, 2004). Such comparisons are valuable, as each approach carries distinct assumptions and sources of measurement error.

The study’s strengths include the large, well-characterized, demographically diverse cohort, which reflects populations commonly enrolled in Alzheimer’s disease and aging studies. The inclusion of participants across the cognitive spectrum, from unimpaired to demented, enhances the real-world applicability of our findings. Moreover, the short inter-scan interval and consistent imaging conditions allowed us to estimate measurement variability with minimal influence from biological brain change.

In summary, the study quantifies expected measurement variability for key Alzheimer’s disease–related structural brain biomarkers, enabling power and sample size calculations for future cross-sectional and longitudinal studies in older adults. The findings further underscore the importance of rigorous quality control and the use of longitudinal processing to achieve optimal measurement precision.

## Figures and Tables

**Table 1:** Estimates of measurement precision for FreeSurfer 7.1 run in the standard cross-sectional mode. Columns show different reliability measures for entorhinal cortex and AD signature thickness, and for hippocampal, lateral ventricle, choroid plexus, and total intracranial volumes.

Measure	Avg (MRI1+MRI2)/2		Diff MRI2−MRI1		|MRI2−MRI1| %Avg		RMSD		ICC (95% CI)	
	Mean	StDev	Mean	StDev	Mean	StDev	Mean	%Avg		

Ento L, mm	3.01	0.31	0.017	0.238	5.90%	5.26%	0.238	7.89%	0.74	(0.64, 0.82)
Ento R, mm	3.11	0.32	0.001	0.234	5.63%	4.92%	0.232	7.46%	0.76	(0.67, 0.84)
Ento avg, mm	3.06	0.28	0.009	0.203	4.92%	4.41%	0.202	6.59%	0.77	(0.68, 0.84)
AD sign, mm	2.55	0.10	0.008	0.054	1.57%	1.44%	0.054	2.13%	0.85	(0.79, 0.90)
Hip L, cm^3^	3.61	0.46	0.030	0.135	3.00%	2.34%	0.137	3.80%	0.96	(0.94, 0.97)
Hip R, cm^3^	3.72	0.47	−0.004	0.118	2.48%	1.97%	0.118	3.17%	0.97	(0.96, 0.98)
Hip Whole, cm^3^	7.33	0.90	0.026	0.213	2.36%	1.72%	0.214	2.91%	0.97	(0.96, 0.98)
Hip/ICV (× 1000)	4.95	0.65	0.023	0.158	2.52%	2.01%	0.159	3.21%	0.97	(0.96, 0.98)
Ven L, cm^3^	18.26	9.89	0.169	0.430	1.83%	1.74%	0.460	2.52%	1.00	(1.00, 1.00)
Ven R, cm^3^	16.49	9.17	0.097	0.588	2.27%	2.80%	0.593	3.60%	1.00	(1.00, 1.00)
Ven whole, cm^3^	34.75	18.71	0.266	0.891	1.85%	1.93%	0.926	2.66%	1.00	(1.00, 1.00)
Ven/ICV (× 1000)	22.82	11.06	0.167	0.627	1.97%	2.04%	0.645	2.83%	1.00	(1.00, 1.00)
Cho L, cm^3^	0.80	0.22	−0.007	0.108	10.73%	8.22%	0.108	13.49%	0.89	(0.84, 0.92)
Cho R, cm^3^	0.81	0.23	−0.028	0.105	10.06%	8.88%	0.108	13.39%	0.89	(0.84, 0.93)
Cho whole, cm^3^	1.61	0.43	−0.035	0.182	8.61%	7.62%	0.184	11.47%	0.91	(0.87, 0.94)
Cho/ICV (× 1000)	1.07	0.23	−0.020	0.128	8.82%	8.23%	0.129	12.03%	0.86	(0.79, 0.90)
ICV, cm^3^	1492	180	−1.03	23.1	0.77%	1.31%	23.2	1.51%	0.99	(0.99, 1.00)

**Table 2:** FreeSurfer 7 reliability, longitudinal mode. See Table 1 notes for explanation of terms.

Measure	Avg (MRI1+MRI2)/2		Diff MRI2−MRI1		|MRI2−MRI1| %Avg		RMSD		ICC (95% CI)	
	Mean	StDev	Mean	StDev	Mean	StDev	Mean	%Avg		

Ento L, mm	2.94	0.28	0.007	0.113	2.96%	2.42%	0.112	3.82%	0.93	(0.89, 0.95)
Ento R, mm	3.06	0.31	0.014	0.111	2.78%	2.37%	0.112	3.65%	0.94	(0.91, 0.96)
Ento avg, mm	3.00	0.26	0.011	0.083	2.09%	1.85%	0.084	2.79%	0.95	(0.93, 0.97)
AD sign, mm	2.60	0.10	0.002	0.046	1.29%	1.23%	0.046	1.77%	0.90	(0.85, 0.93)
Hip L, cm^3^	3.55	0.48	−0.007	0.100	2.27%	1.67%	0.100	2.82%	0.98	(0.97, 0.99)
Hip R, cm^3^	3.65	0.49	−0.009	0.097	2.10%	1.64%	0.097	2.66%	0.98	(0.97, 0.99)
Hip Whole, cm^3^	7.20	0.95	−0.016	0.177	1.95%	1.50%	0.177	2.45%	0.98	(0.98, 0.99)
Hip/ICV (× 1000)	4.87	0.69	−0.009	0.125	1.99%	1.63%	0.125	2.57%	0.98	(0.98, 0.99)
Ven L, cm^3^	17.88	9.88	0.118	0.406	1.65%	1.69%	0.421	2.35%	1.00	(1.00, 1.00)
Ven R, cm^3^	16.40	9.35	0.111	0.420	1.83%	1.91%	0.432	2.63%	1.00	(1.00, 1.00)
Ven whole, cm^3^	34.28	18.86	0.229	0.804	1.69%	1.75%	0.832	2.43%	1.00	(1.00, 1.00)
Ven/ICV (× 1000)	22.50	11.18	0.142	0.502	1.68%	1.59%	0.519	2.31%	1.00	(1.00, 1.00)
Cho L, cm^3^	1.42	0.33	0.003	0.142	6.72%	7.31%	0.141	9.90%	0.92	(0.88, 0.94)
Cho R, cm^3^	1.36	0.30	−0.017	0.132	5.45%	8.09%	0.132	9.73%	0.91	(0.86, 0.94)
Cho whole, cm^3^	2.79	0.60	−0.014	0.251	5.19%	7.37%	0.250	8.98%	0.92	(0.88, 0.94)
Cho/ICV (× 1000)	1.86	0.33	−0.009	0.159	5.13%	6.83%	0.159	8.51%	0.89	(0.84, 0.92)
ICV, cm^3^	1493	179	0	0	0.00%	0.00%	0	0.00%	1.00	(1.00, 1.00)

Tables 1 and 2 Notes:.

Ento L/R/avg – Entorhinal cortex thickness (mm), in the left or right hemisphere, or average, respectively.

AD Sign – AD Signature (mm), cortical thickness averaged across 14 brain regions in the left and right hemisphere (Dickerson et al. Cereb Cortex. 2009;19(3):497–510; PMID: 18632739), including medial temporal; temporal pole; inferior temporal; supramarginal gyrus; superior parietal; precuneus; superior frontal (angular gyrus and inferior frontal sulcus were excluded).

Hip L/R/whole – Hippocampal volume (cm^3^), in the left or right hemisphere, and whole hippocampus (Hip L + Hip R).

Hip/ICV (×1000) – Whole hippocampus volume normalized by ICV (multiplied by 1000).

ICV – Estimated intracranial volume (cm^3^). Note that FreeSurfer does not create an ICV mask but instead estimates ICV from the affine transform of the T1 image to the atlas space. In the reported ICV mean and stdev, only the first three figures are significant, given the magnitude of the random error.

Avg, 0.5*(MRI1+MRI2) – Average of MRI1 and MRI2.

MRI2−MRI1 – Difference between MRI2 and MRI1 (mm, cm^3^, or unitless), mean and stdev across all subjects.

|MRI2−MRI1|, %Avg – Absolute difference expressed as percent average, |MRI2−MRI1|/(0.5*(MRI1+MRI2)), mean and stdev across all subjects.

RMSD, Mean and %Avg – Root mean square difference between MRI2 and MRI1 across all subjects, in respective units (mm, cm^3^, or unitless) or as percent average.

ICC (95% CI) – Intraclass correlation coefficient between MRI1 and MRI2 measurements and 95% confidence interval of ICC.

**Table 3:** Sample size needed to detect within-individual 1% change in various measures (paired t-test power analysis).

	Estimated sample size alpha, two-sided: 0.05, Power: 0.8	
Measure	Cross-sectional	Longitudinal

Ento L, mm	496	122
Ento R, mm	450	103
Ento avg, mm	339	63
AD sign, mm	36	27
Hip L, cm^3^	113	63
Hip R, cm^3^	82	56
Hip Whole, cm^3^	69	50
Hip/ICV (× 1000)	81	54
Ven L, cm^3^	46	43
Ven R, cm^3^	102	54
Ven whole, cm^3^	54	46
Ven/ICV (× 1000)	62	42
Cho L, cm^3^	1433	810
Cho R, cm^3^	1018	700
Cho whole, cm^3^	1018	633
Cho/ICV (× 1000)	1065	552

## Data Availability

The data used in this study contain sensitive human participant information and cannot be made publicly available due to institutional ethical restrictions and participant privacy protections. De-identified data may be made available to qualified researchers upon reasonable request and completion of a data use agreement. Analysis code used in this study is available from the corresponding author upon reasonable request.
